# Keratinocyte Growth Factor-2 Is Protective in Oleic Acid-Induced Acute Lung Injury in Rats

**DOI:** 10.1155/2019/9406580

**Published:** 2019-07-15

**Authors:** S. Tenghao, M. Shenmao, W. Zhaojun, B. Jijia, Z. Wenjie, Z. Wenyan, M. Xigang

**Affiliations:** ^1^Department of Critical Care Medicine, General Hospital of Ningxia Medical University, Yinchuan, Ningxia 750004, China; ^2^Ningxia Medical University, Yinchuan ,Yinchuan, Ningxia 750004, China; ^3^Standardized Training Base, General Hospital of Ningxia Medical University, Yinchuan, Ningxia 750004, China

## Abstract

**Objective:**

The aim of this study was to examine the role of keratinocyte growth factor-2 (KGF-2) in oleic acid-induced acute lung injury (ALI) in rats.

**Methods:**

Forty-five healthy adult male Sprague Dawley rats were divided into 3 groups. Rat ALI model was established by injection of 0.01 mL/kg oleic acid into the tail vein. Rats in the control group were injected with the same amount of normal saline (NS). In the ALI + KGF-2 group, 5 mg/kg of KGF-2 was instilled into the airway of rats 72 hours before the model preparation, and the control group and the ALI model group were instilled with the same amount of NS. The lung permeability index (LPI) and lung wet/dry weight (W / D) ratios were measured 8 hours after the model preparation. The permeability of pulmonary microvascular endothelium was evaluated by Evans blue leakage test. Histopathological changes were observed under light microscope and the ALI pathology score (LIS) was calculated. Ultrastructural changes of lung tissue were observed under electron microscope. The apoptosis was detected by TUNEL assay. The expression of Claudin-5, ZO-1, and VE Cadherin in lung tissue was qualitatively and quantitatively analyzed by immunohistochemistry, Western Blot, and qRT-PCR, respectively.

**Results:**

The ALI model group had severe lung injury and obvious pathological changes, including alveolar septal thickening and inflammatory cell infiltration. TUNEL assay showed that the apoptosis of ALI group was significantly increased. The LIS score, lung W/D ratio, LPI, and Evans blue leakage were significantly higher than those in the control group; electron microscopy showed that the alveolar-capillary barrier was severely damaged in the ALI group. Compared with the control group, the expression of Claudin-5, ZO-1, and VE cadherin in the lung tissue of the ALI model group was significantly attenuated. After pretreatment with KGF-2, the degree of lung tissue damage was significantly reduced and the pathological changes were significantly improved. TUNEL assay showed that the apoptosis of ALI group was decreased. Lung W/D ratio, LPI, and Evans blue leakage decreased; electron microscopy showed that the alveolar-capillary barrier of ALI group recovered significantly. Compared with the ALI model group, the expression of Claudin-5, ZO-1, and VE cadherin in the lung tissue of the KGF-2 pretreatment group increased.

**Conclusion:**

The results indicate that KGF-2 may attenuate oleic acid-induced ALI in rats by maintaining the pulmonary microvascular endothelial barrier, which is an effective ALI preventive measure.

## 1. Introduction

Diffuse alveolar-capillary injury is considered to be an important cause of acute lung injury (ALI)/acute respiratory distress syndrome (ARDS) [1–3]. After alveolar-capillary injury, pulmonary vascular and pulmonary interstitial fluid exchange disorders lead to osmotic pulmonary edema, which ultimately leads to refractory hypoxemia and respiratory distress [2, 4]. The diffuse pulmonary microvascular endothelial cell injury caused the destruction of pulmonary microvascular endothelial barrier, and the interstitial edema caused by increased permeability is a very important pathological change of ALI [5]. Therefore, maintaining the integrity of the pulmonary microvascular endothelial barrier structure and reducing pulmonary microvascular permeability are the keys to ALI treatment. However, no effective drug has been found to protect the pulmonary microvascular endothelial barrier and reduce the mortality of ALI [6, 7].

Keratinocyte growth factor-2 (KGF-2) binds to its receptors FGFR2-IIIb (KGFR) and FGFR1III-b, promoting epithelial cell growth, differentiation, and migration [8–12]. At the same time, it was found that KGF-2 can increase angiogenesis and maintain capillary barrier [13]. However, the specific role of KGF-2 in protecting the pulmonary microvascular endothelial barrier remains unclear. Therefore, the purpose of this study was to evaluate alveolar-capillary changes in KGF-2 ALI and to explore its underlying mechanisms.

## 2. Materials and Methods

### 2.1. Animals

Forty-five male Sprague Dawley rats (the Experimental Animal Center of Ningxia Medical University, Yinchuan, China), 6 to 7 weeks old, weighing 220 ± 20 g, were housed in animal facilities at the General Hospital of Ningxia Medical University with clean, controlled temperature and independent ventilation. These rats are free to access food and water. All experimental protocols were approved by the Ethics Committee of the General Hospital of Ningxia Medical University.

### 2.2. Grouping and Animal Handling

All rats were randomly divided into 3 groups: control group, ALI group, and ALI+KGF-2 group. Animal ALI model was prepared by injecting oleic acid 0.1 mL/kg (Sigma-Aldrich, St.Louis, MO, USA) into the tail vein of rats; 0.1 mL/kg saline was administered to the control group. In the ALI+KGF-2 group, the 18G catheter connected to the 1ml syringe was inserted into the trachea 72 hours before the model preparation, and the 5mg/kg dose of KGF-2(recombinant human KGF-2, 5 mg/kg in 0.5 ml NS; Wenzhou Medical University School of Pharmacy, Wenzhou, China) was instilled into the lungs of the rats. In the control group and the ALI group, 5 mg/kg NS was instilled. Rats were anesthetized by intraperitoneal injection of 2% sodium pentobarbital (60 mg/kg).

### 2.3. Lung Wet-to-Dry Weight Ratio (W/D) and Lung Permeability Index (LPI)

The rats were killed after 8 h of the model preparation. Blood was collected from the abdominal aorta of the rat and bronchoalveolar lavage fluid (BALF) was collected. The rat chest wall was cut and the bilateral lungs were exposed. The right hilum is then ligated and the neck is cut open to expose the trachea. A 5Fr plastic endotracheal catheter (approximately 2 mm in diameter and 1.2 mm in inner diameter; Shenzhen Corigo Medical Instrument Co., Ltd., Shenzhen, China) was inserted into the trachea and fixed with threads. 10 ml of normal saline was injected into the catheter, and about 8 mL of bronchoalveolar lavage fluid was collected. The BALF double gauze was then filtered and centrifuged at 4000 r/min for 10 minutes at 4°C, and the supernatant was collected. BALF was quantified using a BCA protein assay kit (Nanjing KeyGen Biotech Co., Ltd., Nanjing, China). LPI = protein concentration in BALP/plasma protein concentration. The upper lung lobe of the right lung was collected and rinsed with NS. After removing water and blood from the surface with a filter paper and the upper lobe was weighed and dried at 80°C in a 202-2 electrothermal oven (Experimental Instrument Co., Ltd., Shanghai, China). The lung W/D weight ratio was calculated from 72 hours to constant weight.

### 2.4. Lung Morphometry Analyses

After sacrifice, the rats were harvested and the lower lobe of the right lung was collected and fixed in 4% paraformaldehyde for 24 h at 4°C. After dehydration and transparency, they were embedded in paraffin and cut into 4 *μ*m sections, followed by hematoxylin-eosin (HE) dyeing. Lung injury was scored [14] according to the following variables: 1 point of alveolar septal thickening; 1 point of alveolar hemorrhage; 1 point of fibrin deposition in alveolar; 1 point of inflammatory cell infiltration in alveolar, the total score is LIS score.

### 2.5. TUNEL Apoptosis Detection

The lung tissue was embedded in paraffin and cut into 4 *μ*m thick sections. The sections were then stained with TUNEL Apoptosis Assay Kit (Nanjing KeyGen Biotech Co., Ltd., Nanjing, China). The number of apoptotic cells was obtained. It is used to reflect the severity of apoptosis in lung epithelial cells and lung endothelial cells.

### 2.6. Lung Microvascular Permeability

Lung microvascular permeability was performed with Evans blue dye (EBD) method [15]. Evans blue dye (Sigma-Aldrich, St. Louis, MO, USA) (20 mg / kg) was fused in 1 mL of NS and injected through the tail vein 8 hours after model preparation. After 30 minutes, 100 mL of heparinized saline was injected into the right ventricle of the heart to remove the dye from the pulmonary blood vessels. 100 mg lung tissue from the right lung lobe was incubated in formamide (Sigma-Aldrich, St. Louis, MO, USA) for 24 hours at 60°C. It was then measured at 630 nm.

### 2.7. Transmission Electron Microscopy

Lung tissue samples were washed with phosphate buffered saline (PBS) and fixed with 2% glutaraldehyde for 2 hours. The lung tissue was then fixed with 1% osmium tetroxide. The tissue is then dehydrated with graded ethanol and embedded in propylene oxide and cut into ultrathin sections and stain with uranyl acetate and lead citrate. Finally, the ultrastructure of lung tissue was examined by transmission electron microscopy (Hitachi H-7650, Hitachi, Naka, Japan).

### 2.8. Immunohistochemistry

The lung tissue sections were routinely dewaxed and hydrated; Claudin-5 antibody (1:200; ab131259; Abcam), ZO-1 antibody (1:300; ab96587; Abcam), and VE cadherin antibodies (1:300; ab231227; Abcam) were incubated overnight at 4°C and negative controls were incubated with PBS. Sections were covered with 3,3'-diaminobenzidine (DAB) tetrahydroxychloride and counterstained with hematoxylin. Under light microscopy, the tissue was dark brown for protein positive expression, and no staining or staining was negative or weakly positive. The average optical density (AOD) of the positive expression was analyzed by IPP software = integrated optical density (IOD)/positive expression area.

### 2.9. Western Blot Analyses

Lung tissues were homogenized and 40 ug of protein was electrophoresed. Membranes were exposed overnight at 4°C to Claudin-5 antibody (1:150), ZO-1 antibody (1:200), VE cadherin antibodies (1:500), and rabbit anti-GAPDH antibody (1:500; ab9485; Abcam) as loading control.

### 2.10. Quantitative Real-Time Polymerase Chain Reaction Analyses

Total RNA was extracted using TRIzol® reagent (Invitrogen Inc., Carlsbad, CA, USA) from lung tissues. First-strand cDNA was synthesized by using a RevertAid™ first-strand cDNA synthesis kit (Thermo Scientific Inc., Wilmington, DE, USA). Quantitative real-time PCR was performed using Maxima SYBR Green qPCR Master Mix (Thermo Fisher Scientific Inc, Beijing, China) with the CFX96 Real-Time PCR Detection System using 2ul of cDNA in a 25ul reaction volume. Reactions were incubated for 30 seconds at 95°C, followed by 40 cycles of 95°C for 5 seconds and 60°C for 30 seconds. Real-time polymerase chain reaction was performed using primers for the examined transcripts. The specific primer used for GADPH is as follows: Forward 5'-CCGTATCGGACGCCTGGTTA-3', Reverse 5'-CAGTGATGGATGGACTGTGGT-3', claudin-5 was: Forward 5'-CAGCGTTGGAAATTCTGGGTC-3', Reverse 5'-ACACTTTGCATTGCATGTGCC-3', zo-1 was:Forward5'-AGTATAATTATCCCACAAGGAGCCA-3', Reverse 5'-TTTAGGGTCACAGTGTGGCAA-3', VE Cadherin was:Forward 5'-TTCAGGCCCCTTAAATATCCAG-3', Reverse 5'-CCAGAGAGAATACTAGTCTCGC-3'.

### 2.11. Statistical Analysis

All measurement data are expressed as mean±standard deviation. Nonnormally distributed data is transformed into a normal distribution after logarithm analysis. One-way analysis of variance (ANOVA) was used to detect differences in normal distribution data between groups, and the pairwise comparison between groups was performed by LSD test. All statistical analyses were performed using statistical software SPSS 22.0, and* P* < 0.05 was considered statistically significant. Eight independent experiments were performed in this study.

## 3. Results

### 3.1. Effect of KGF-2 on Morphological Changes of Lung Tissue Induced by Oleic Acid in ALI Rats

**Figure 1:**
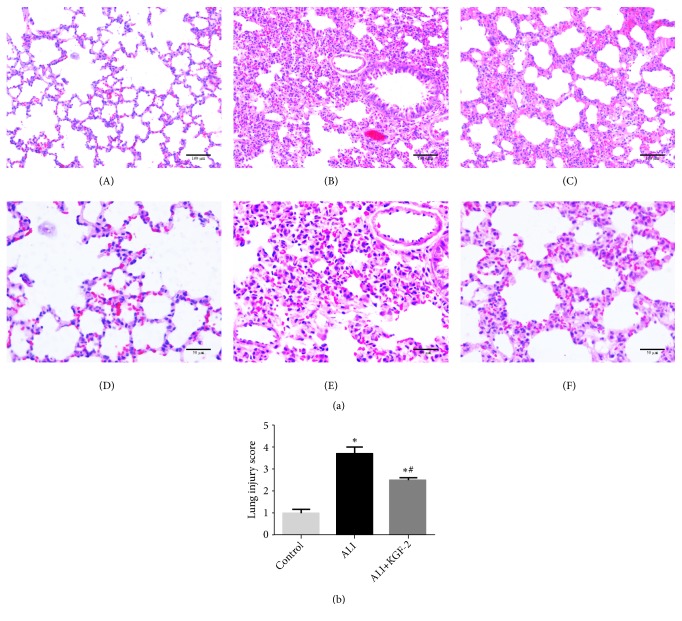
(a) Pathological changes of lung tissue in each group were observed under light microscope. (A) The alveolar structure of the control group was intact (middle magnification); (D) there was no obvious inflammatory cell infiltration in the control group and no significant change in the pulmonary interstitial (high magnification); (B) alveolar septal thickening in the ALI group, alveolar fusion (middle magnification); (E) inflammatory cells in the ALI group were extensively infiltrated and a large amount of protein exuded (high magnification); (C) mild fusion of alveolus in the ALI+KGF-2 group (middle magnification); (F) a small amount of inflammatory cell infiltration in ALI+KGF-2 group and a small amount of protein exudation (high magnification) HE staining. (b) Quantitative analysis of lung injury scores in each group (*∗*< 0.01 versus control group, #< 0.01 versus ALI group).

HE staining was used to assess pathological changes in lung tissue (Figure 1(a)). The control group showed intact and clear alveolar structure and no obvious thickening of the alveolar septum. No obvious hyperemia and edema were observed. No obvious inflammatory cell infiltration was observed (Figures 1(A) and 1(D)). In the ALI group, the alveolar space of the lung tissue was thickened, the alveolar space was reduced, and a large number of inflammatory cells accumulated in the alveolar space, and the interstitial lung was congested (Figures 1(B) and 1(E)). In the ALI+KGF-2 group, the pathological changes of the lung tissue were alleviated, the pulmonary congestion and edema were alleviated, and the inflammatory cell infiltration of the alveolar and interstitial parts was reduced (Figures 1(C) and 1(F)). The LIS value of the ALI group was higher than that of the control group (*P* <0.01). Compared with the ALI group, the LIS of the ALI+KGF-2 group was lower (*P* <0.01), but higher than the control group LIS (*P* <0.01) (Figure 1(b)).

**Figure 2:**
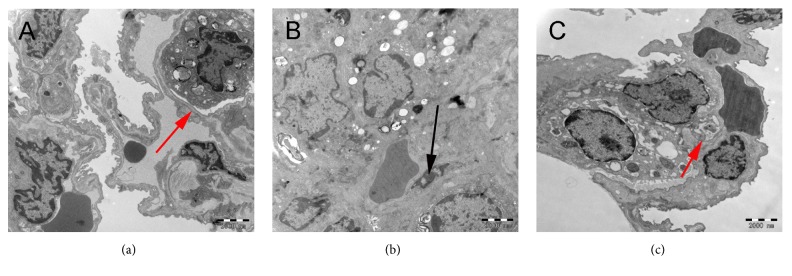
Ultrastructural changes in lung tissue of rats in each group under transmission electron microscopy (10,000 times). (a) Control alveolar-capillary barrier integrity (red arrow); (b) alveolar-capillary barrier was severely impaired in ALI group and alveolar type II epithelial cells were degenerated, endothelial cell apoptosis (black arrow) and basement membrane exposure; (c) ALI+KGF-2 group reduced damage; alveolar-capillary barrier was basically intact (red arrow).

Transmission electron microscopy (TEM) was used to evaluate the ultrastructural changes of lung tissue. The lung microvascular endothelial cells and basement membrane were intact in the control group. The structure of alveolar type II epithelial cells was regular, the nucleus was obvious, the cytoplasm was uniform, and the eosinophilic corpuscles were matured to varying degrees (Figure 2(a)). Compared with the control group and the ALI+KGF-2 group, the alveolar type II epithelial cells in the ALI group were degenerated and destroyed, and the eosinophilic lamellar bodies were vacuolated to varying degrees, and the mitochondrial mites dissolved and were destroyed or even disappeared, resulting in vacuolization, pulmonary microvascular endothelial cell apoptosis, basement membrane destruction, loose, and chromatin accumulation (Figure 2(b)). The morphology of alveolar type II epithelial cells in ALI+KGF-2 group was almost normal, the number of eosinophilic corpuscles increased, vacuolization decreased, microvascular endothelial cells were slightly swollen, the morphology was normal, and the basement membrane was intact (Figure 2(c)).

### 3.2. Effect of KGF-2 on Oleic Acid-Induced Apoptosis in Lung Tissue of ALI Rats

**Figure 3:**
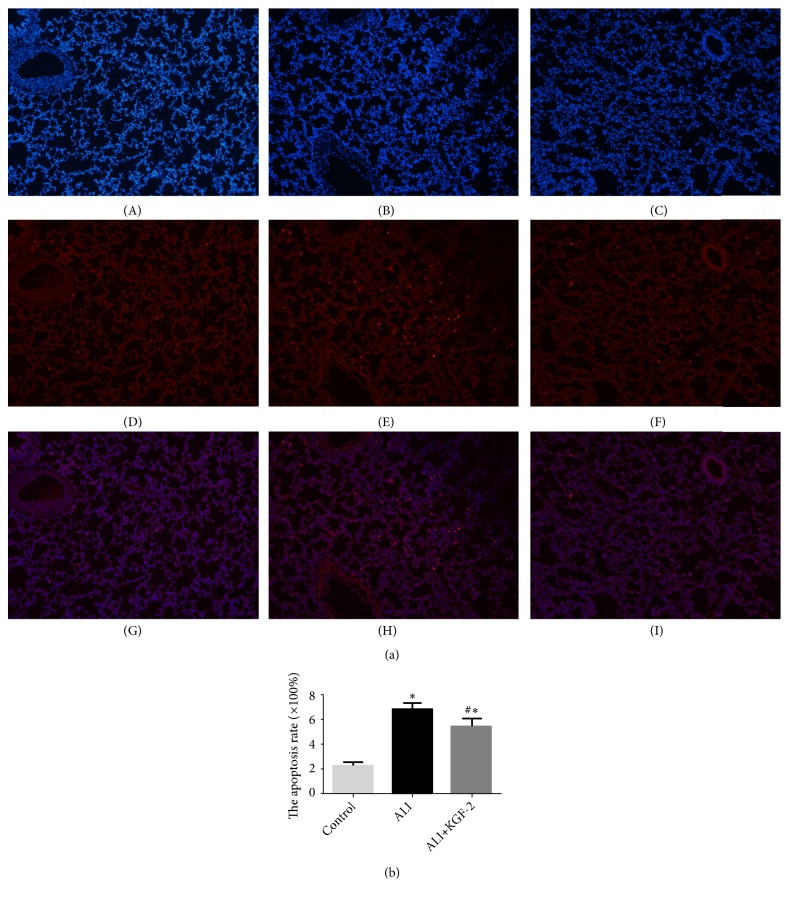
(a) Changes in apoptosis of lung tissue in each group. (A) DAPI in the control group; (B) DAPI in the ALI group; (C) DAPI in the ALI+KGF-2 group; (D) number of apoptotic cells in the control group; apoptotic cells were sparse and scattered; (E) apoptotic cells in ALI group, diffuse distribution of apoptotic cells; (F) number of apoptotic cells in ALI+KGF-2 group, scattered number of apoptotic cells; (G) control group mixed images; (H) mixed images of ALI group; (I) mixed images of ALI+KGF-2 group medium magnification. (b) Statistical analysis of apoptosis of lung tissue of each group (*∗*< 0.01 versus control group, #<0.01 versus ALI group).

Apoptosis of pulmonary microvascular endothelial cells and epithelial cells impairs blood gas barrier integrity and leads to increased lung permeability. The effect of KGF-2 on apoptosis was examined by TUNEL. In the control group, scattered apoptotic cells were scattered between the lung tissues (Figure 3(D)). Compared with the control group and ALI+KGF-2, the number of apoptotic cells in the ALI group increased significantly, especially in endothelial cells (Figure 3(E)). After KGF-2 intervention, the number of apoptotic cells decreased, but it was still higher than the control group (Figure 3(F)). The results showed that there are differences between the three groups and the comparison between the two groups (all* P*< 0.01) (Figure 3(b)).

### 3.3. Effect of KGF-2 on Vascular Permeability Induced by Oleic Acid in ALI Rats

**Figure 4:**
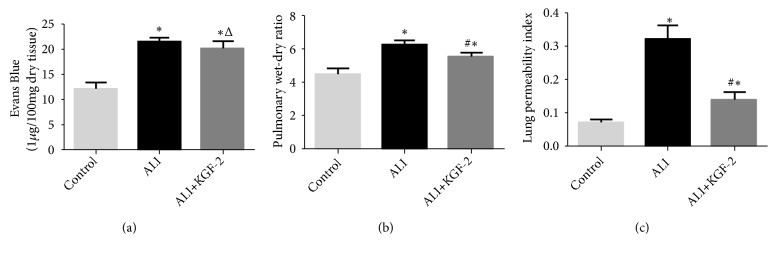
Shows a significant increase in EB content, W/D value, and LPI value in the ALI group compared with the control group and improved after KGF-2 intervention. (a) Evans blue content in the lung. (b) Lung wet-to-dry weight ratio. (c) Lung permeability index (*∗*< 0.01 versus control group; #<0.01 versus ALI group; Δ< 0.05 versus ALI group ).

W/D ratio, LPI, and Evans blue leakout experiments were used to assess changes in pulmonary vascular permeability. Compared with the control group, the W/D ratio and LPI of the ALI group were significantly increased (*P*<0.01). After KGF-2 pretreatment, the lung W/D ratio and LPI were significantly lower than those of the ALI model group (both* P*< 0.01) (Figures 4(b) and 4(c)). Compared with the control group, the EB content in the lung tissue of the ALI group was significantly increased (*P *<0.01), and the EB content was decreased after KGF-2 intervention (*P* <0.05) (Figure 4(a)).

### 3.4. Effects of KGF-2 on the Expression of Claudin-5, ZO-1, and VE Cadherin in Lung Tissue of ALI Rats Induced by Oleic Acid

**Figure 5:**
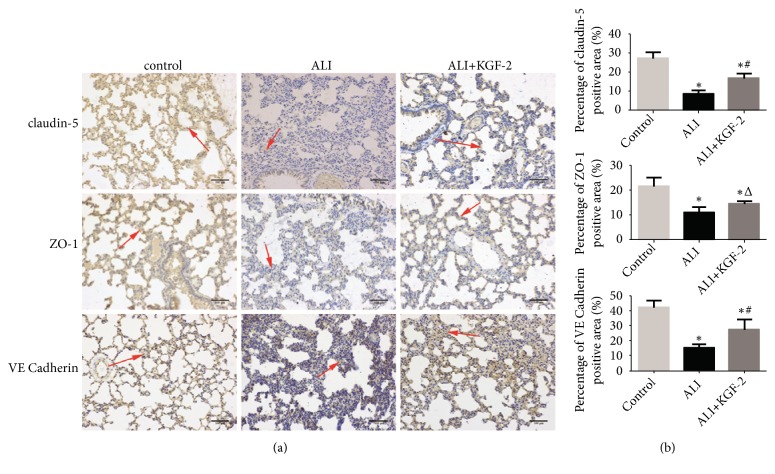
Immunohistochemical staining to determine Claudin-5, ZO-1, and VE cadherin expression in rat lung tissue. (a) Claudin-5 expression in the lung tissue of the control group was strongly positive (dark brown); ALI group Claudin-5 expression was negative in lung tissue (light yellow); Claudin-5 expression was weakly positive (brown) in lung tissue of ALI+KGF-2 group; ZO-1 expression in lung tissue of control group strong positive (dark brown); ZO-1 expression was negative in the lung tissue of ALI group (light yellow) and showed that ZO-1 expression was weakly positive in lung tissue of ALI+KGF-2 group (brown yellow); VE cadherin expression was strongly positive in the lung tissue of the control group (dark brown); VE cadherin expression was negative in the lung tissue of the ALI group (light yellow); ALI+KGF-2 group lung tissue VE cadherin expression is weakly positive (brownish yellow). Medium magnification. Positive expression of brown stained strip is indicated by the arrow; (b) statistical analysis of positive expression of each group (*∗*< 0.01 versus control group, #<0.01 versus ALI group, and Δ< 0.05 versus ALI group ).

Claudin-5, ZO-1, and VE cadherin is mainly expressed at the junction of cells and the cell membrane. Immunohistochemistry showed that the positive expression of Claudin-5, ZO-1, and VE cadherin was significantly decreased in the ALI group compared with the control group. After KGF-2 pretreatment, the positive expression of Claudin-5, ZO-1, and VE cadherin in rat lung tissue was significantly higher than that in ALI group (Figure 5).

**Figure 6:**
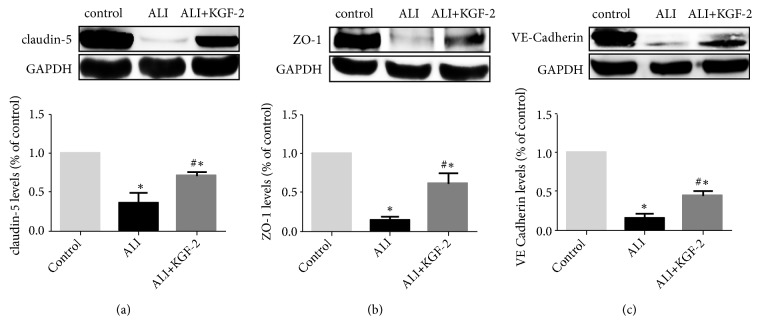
Western blot analysis of Claudin-5, ZO-1 and VE Cadherin expression in rat lung tissue. (a) Claudin-5 protein polyacrylamide gel electrophoresis and protein expression level (*∗*< 0.01 versus control group; #< 0.01 versus ALI Group); (b) ZO-1 protein polyacrylamide gel electrophoresis and protein expression level (*∗*< 0.01 versus control group; #< 0.01 versus ALI Group); (c) VE cadherin protein polyacrylamide gel electrophoresis and protein expression level (*∗*< 0.01 versus control group; #< 0.01 versus ALI Group).

Western Blot was used to evaluate the changes of Claudin-5, ZO-1, and VE cadherin in the lung tissues of each group. Compared with the control group, the expression levels of Claudin-5, ZO-1, and VE Cadherin in lung tissue of ALI group were significantly decreased (all* P*<0.01). After KGF-2 pretreatment, the expression levels of Claudin-5, ZO-1, and VE cadherin in lung tissue were significantly higher than those in ALI model group (all* P*<0.01), but still higher than the control group (all* P*<0.01) (Figure 6).

**Figure 7:**
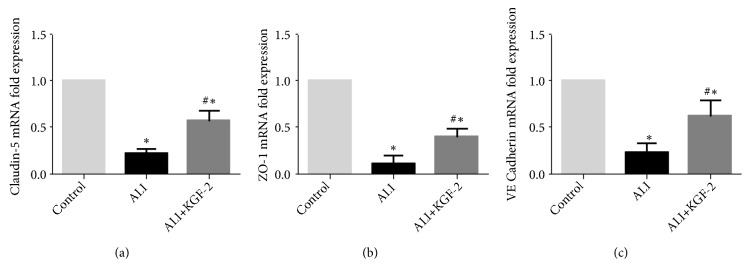
qRT-PCR analysis of Claudin-5, ZO-1 and VE cadherin expression in rat lung tissue. (a) Detection of Claudin-5 mRNA expression levels in rat lung tissue by qRT-PCR assay (*∗*< 0.01 versus control group; #<0.01 versus ALI group); (b) detection of ZO-1 mRNA expression levels in rat lung tissue by qRT-PCR assay (*∗*< 0.01 versus control group; #<0.01 versus ALI group); (c) detection of VE Cadherin mRNA expression levels in rat lung tissue by qRT-PCR assay (*∗*< 0.01 versus control group; #<0.01 versus ALI group).

qRT-PCR was used to detect mRNA expression levels in lung tissue. The expression levels of Claudin-5, ZO-1, and VE cadherin mRNA in lung tissue of ALI group were significantly lower than those in control group (all* P* < 0.01), and the expression levels of Claudin-5, ZO-1, and VE cadherin mRNA after KGF-2 pretreatment. There was an increase (all* P* < 0.01) (Figure 7).

## 4. Discussion

In this experiment, 0.01 ml/kg oleic acid was injected through the tail vein, and ALI was induced in rats after 8 hours. The study found that keric acid-induced rat ALI can be improved by KGF-2 pretreatment. Pretreatment with KGF-2 can improve histological changes, reduce apoptosis, and reduce lung tissue edema. The protective effect of KGF-2 was further confirmed by observing changes in the expression of related proteins on the pulmonary microvascular endothelial barrier. It is indicated that KGF-2 has a protective effect on oleic acid-induced ALI in rats by restoring the pulmonary microvascular endothelial barrier.

Intravenous injection of oleic acid for 8h can rapidly reproduce the basic characteristics of ALI in the early stage; that is, alveolar-capillary permeability changes, pulmonary microvascular endothelial barrier is destroyed, and protein-rich liquid penetrates into alveolar and pulmonary interstitial, affecting Gas exchange [16–19]. At present, there is no relevant drug treatment plan, and finding new treatments is of great significance for improving ALI.

KGF-2, also known as fibroblast growth factor-10 (FGF-10), shares homology with KGF and plays an important role in lung development, lung inflammation, and repair [20]. Nana Feng et al. found that KGF-2 can inhibit bacterial infection of* P. aeruginosa* pneumonia in mice and has a protective effect on the lung epithelial barrier [21]. Jun She et al. found that KGF-2 can significantly improve the survival rate and lung injury of rats with high altitude pulmonary edema and has protective effects on epithelial and endothelial stress [13]. Jing Bi et al. found that KGF-2 can protect rat ALI caused by ventilator by reducing inflammation and restoring alveolar surfactants [22]. Lin Tong et al. found that KGF-2 stimulates the proliferation of alveolar type II epithelial cells by inhibiting inflammatory response and inhibiting lipopolysaccharide-induced ALI in rats [23]. Xiaocong Fang et al. found that intratracheal instillation of KGF-2 can reduce lung tissue edema and leukocyte infiltration, thereby protecting lung injury induced by ischemia-reperfusion [24]. However, the protective effect of KGF-2 on oleic acid-induced ALI, especially the protection of pulmonary microvascular endothelial barrier, is still not fully understood. In this study, we started from alveolar-capillary permeability and found that KGF-2 can restore the pulmonary microvascular endothelial barrier and reduce the tight junctional destruction between cells.

The pathogenesis of ALI is very complex, and alveolar-capillary permeability changes are the main pathological changes. In its essence, increased microvascular permeability is the underlying cause [25, 26]. The tight junctional destruction between cells is a key pathological factor leading to increased alveolar-capillary permeability [27–29]. The tight junction is a network-like closed structure located at the apical end of the cell, which is mainly composed of the Claudin protein family and the related protein zo-1 [30–33]. The ability to connect and close cell gaps determines the permeability of the intercellular space [34–36]. Armstrong SM et al. found that Claudin-5 expression was significantly reduced in the study of infection-induced damage to the pulmonary microvascular endothelial barrier function [37]. Deissler HL et al. found that elevated barrier permeability of retinal endothelial cells in diabetic retinopathy is associated with decreased expression of ZO-1 [38]. In addition, studies have found that hypoxia can reduce the expression of ZO-1 and Claudin-5, which in turn leads to the destruction of vascular endothelial barrier function and increases the permeability of vascular endothelial cells [39]. Vascular endothelial cell cadherin (VE cadherin) is an important component of adhesion between endothelial cells and plays a key role in maintaining vascular integrity [40, 41]. Claudin-5, ZO-1, and VE cadherin can largely reflect the integrity of the pulmonary microvascular endothelial barrier.

After pretreatment with KGF-2, the histological structure of ALI in rats induced by oleic acid improved, and the number of apoptosis after KGF-2 pretreatment was reduced by TUNEL staining. From a macroperspective, KGF- 2 has protective effect on ALI induced by oleic acid. Through the lung dry-to-wet ratio, lung permeability barrier, and Evans blue leakage test, it was confirmed that KGF-2 pretreatment significantly reduced lung tissue edema, decreased tissue leakage, and reduced alveolar-capillary barrier damage. Ultrastructural changes were observed under electron microscope, and it was further confirmed that the ALI induced alveolar-capillary barrier of ALI was improved in rats after KGF-2 pretreatment. It is indicated that KGF-2 has protective effect on ALI alveolar-capillary barrier induced by oleic acid. Qualitative and quantitative analysis of Claudin-5, ZO-1, and VE cadherin protein showed that the expression of Claudin-5, ZO-1, and VE cadherin protein decreased in different degrees when oleic acid-induced ALI; KGF- 2 After pretreatment, the expression of Claudin-5, ZO-1, and VE cadherin protein recovered to different extents. It indicated that KGF-2 has protective effect on oleic acid-induced microvascular endothelial barrier in rats.

In this study, rats in the control group were instilled with a certain proportion of normal saline in the airway, which may cause very mild lung injury, but the results of the study confirmed statistically significant between the three groups, further indicating that it is statistically significant compared with normal rats. The next step in this study will be to further investigate the protective effect of KGF-2 on the microvascular endothelial barrier of ALI from the in vitro cell level.

In short, this study confirmed the protective effect of KGF-2 pretreatment on oleic acid-induced ALI in rats, and its mechanism is related to the recovery of pulmonary microvascular endothelial barrier. Based on this study, KGF-2 was aerosolized into the lungs to repair the pulmonary microvascular endothelial barrier, providing new theoretical guidance for the treatment of ALI from the root.

## 5. Conclusions

The present experiments indicate that the protective effect of KGF-2 pretreatment on oleic acid-induced ALI in rats, and its mechanism is related to the recovery of pulmonary microvascular endothelial barrier.

## Data Availability

The data (photos) used to support the findings of this study are available from the corresponding author upon request.

## Supplementary Materials

Supplementary Materials This support material focuses on disease ALI/ARDS and target location for intervention in this study as well as supplemental experiments (ELISA for TNF-a/IL-10).

## References

[1] Chen B., Yang Z., Yang C. (2018). A self-organized actomyosin drives multiple intercellular junction disruption and directly promotes neutrophil recruitment in lipopolysaccharide-induced acute lung injury. *The FASEB Journal*.

[2] Herold S., Gabrielli N. M., Vadász I. (2013). Novel concepts of acute lung injury and alveolar-capillary barrier dysfunction. *American Journal of Physiology-Lung Cellular and Molecular Physiology*.

[3] Kim Y.-Y., Lee S., Kim M.-J. (2017). Tyrosol attenuates lipopolysaccharide-induced acute lung injury by inhibiting the inflammatory response and maintaining the alveolar capillary barrier. *Food and Chemical Toxicology*.

[4] Bhattacharya J., Matthay M. A. (2013). Regulation and repair of the alveolar-capillary barrier in acute lung injury. *Annual Review of Physiology*.

[5] Yang Y., Hu S., Xu X. (2016). The vascular endothelial growth factors-expressing character of mesenchymal stem cells plays a positive role in treatment of acute lung injury* in vivo*. *Mediators of Inflammation*.

[6] Sweeney R., Griffiths M., McAuley D. (2013). Treatment of acute lung injury: current and emerging pharmacological therapies. *Seminars in Respiratory and Critical Care Medicine*.

[7] Impellizzeri D., Bruschetta G., Esposito E., Cuzzocrea S. (2015). Emerging drugs for acute lung injury. *Expert Opinion on Emerging Drugs*.

[8] Yu L. S., Li X. B., Fan Y. Y. (2017). Expression of KGF-1 and KGF-2 in skin wounds and its application in forensic pathology. *The American Journal of Forensic Medicine and Pathology*.

[9] Saksena S., Priyamvada S., Kumar A. (2013). Keratinocyte growth factor-2 stimulates P-glycoprotein expression and function in intestinal epithelial cells. *American Journal of Physiology-Gastrointestinal and Liver Physiology*.

[10] Wang J., Chen H., Wang Y. (2016). Therapeutic efficacy of a mutant of keratinocyte growth factor-2 on trinitrobenzene sulfonic acid-induced rat model of Crohn's disease. *American Journal of Translational Research*.

[11] Shi X., Liu H., Li S., Xu H. (2018). Keratinocyte growth factor protects endometrial cells from oxygen glucose deprivation/re-oxygenation via activating Nrf2 signaling. *Biochemical and Biophysical Research Communications*.

[12] Cai J., Dou G., Zheng L. (2015). Pharmacokinetics of topically applied recombinant human keratinocyte growth factor-2 in alkali-burned and intact rabbit eye. *Experimental Eye Research*.

[13] She J., Goolaerts A., Shen J. (2012). KGF-2 targets alveolar epithelia and capillary endothelia to reduce high altitude pulmonary oedema in rats. *Journal of Cellular and Molecular Medicine*.

[14] Su X., Song Y., Jiang J., Bai C. (2004). The role of aquaporin-1 (AQP1) expression in a murine model of lipopolysaccharide-induced acute lung injury. *Respiratory Physiology & Neurobiology*.

[15] Moitra J., Sammani S., Garcia J. G. (2007). Re-evaluation of Evans Blue dye as a marker of albumin clearance in murine models of acute lung injury. *Translational Research*.

[16] Chen S., Zheng S., Liu Z. (2015). Endogeous sulfur dioxide protects against oleic acid-induced acute lung injury in association with inhibition of oxidative stress in rats. *Laboratory Investigation*.

[17] Zhou M., Osanai K., Kobayashi M. (2014). Adenovector-mediated gene transfer of lysophosphatidylcholine acyltransferase 1 attenuates oleic acid–induced acute lung injury in rats. *Critical Care Medicine*.

[18] Chen H. I., Hsieh N., Kao S. J., Su C. (2008). Protective effects of propofol on acute lung injury induced by oleic acid in conscious rats. *Critical Care Medicine*.

[19] Davidson K. G., Bersten A. D., Barr H. A., Dowling K. D., Nicholas T. E., Doyle I. R. (2000). Lung function, permeability, and surfactant composition in oleic acid-induced acute lung injury in rats. *American Journal of Physiology-Lung Cellular and Molecular Physiology*.

[20] Ware L. B., Matthay M. A. (2002). Keratinocyte and hepatocyte growth factors in the lung: Roles in lung development, inflammation, and repair. *American Journal of Physiology-Lung Cellular and Molecular Physiology*.

[21] Feng N., Wang Q., Zhou J. (2016). Keratinocyte growth factor-2 inhibits bacterial infection with Pseudomonas aeruginosa pneumonia in a mouse model. *Journal of Infection and Chemotherapy*.

[22] Bi J., Tong L., Zhu X. (2014). Keratinocyte growth factor-2 intratracheal instillation significantly attenuates ventilator-induced lung injury in rats. *Journal of Cellular and Molecular Medicine*.

[23] Tong L., Bi J., Zhu X. (2014). Keratinocyte growth factor-2 is protective in lipopolysaccharide-induced acute lung injury in rats. *Respiratory Physiology & Neurobiology*.

[24] Fang X., Wang L., Shi L. (2014). Protective effects of keratinocyte growth factor-2 on ischemia–reperfusion–induced lung injury in rats. *American Journal of Respiratory Cell and Molecular Biology*.

[25] Gill S. E., Rohan M., Mehta S. (2015). Role of pulmonary microvascular endothelial cell apoptosis in murine sepsis-induced lung injury in vivo. *Respiratory Research*.

[26] Müller-Redetzky H. C., Kummer W., Pfeil U. (2012). Intermedin stabilized endothelial barrier function and attenuated ventilator-induced lung injury in mice. *PLoS ONE*.

[27] Shao P., Zhu J., Ding H. (2018). Tripterygium hypoglaucum (Levl.) Hutch attenuates oleic acid-induced acute lung injury in rats through up-regulating claudin-5 and ZO-1 expression. *International Journal of Clinical and Experimental Medicine*.

[28] Gu C., Liu M., Zhao T., Wang D., Wang Y. (2015). Protective role of p120-catenin in maintaining the integrity of adherens and tight junctions in ventilator-induced lung injury. *Respiratory Research*.

[29] Filipczak P. T., Senft A. P., Seagrave J. (2015). NOS-2 inhibition in phosgene-induced acute lung injury. *Toxicological Sciences*.

[30] Schlingmann B., Molina S. A., Koval M. (2015). Claudins: gatekeepers of lung epithelial function. *Seminars in Cell & Developmental Biology*.

[31] Van Itallie C. M., Anderson J. M. (2014). Architecture of tight junctions and principles of molecular composition. *Seminars in Cell & Developmental Biology*.

[32] Krug S. M., Schulzke J. D., Fromm M. (2014). Tight junction, selective permeability, and related diseases. *Seminars in Cell & Developmental Biology*.

[33] Lerner A., Matthias T. (2015). Changes in intestinal tight junction permeability associated with industrial food additives explain the rising incidence of autoimmune disease. *Autoimmunity Reviews*.

[34] Koval M. (2013). Claudin heterogeneity and control of lung tight junctions. *Annual Review of Physiology*.

[35] Caron T. J. (2015). Tight junction disruption: *Helicobacter pylori* and dysregulation of the gastric mucosal barrier. *World Journal of Gastroenterology*.

[36] Bäsler K., Bergmann S., Heisig M., Naegel A., Zorn-Kruppa M., Brandner J. M. (2016). The role of tight junctions in skin barrier function and dermal absorption. *Journal of Controlled Release*.

[37] Armstrong S. M., Wang C., Tigdi J. (2012). Influenza infects lung microvascular endothelium leading to microvascular leak: role of apoptosis and claudin-5. *PLoS ONE*.

[38] Deissler H. L., Deissler H., Lang G. K., Lang G. E. (2013). VEGF but not PlGF disturbs the barrier of retinal endothelial cells. *Experimental Eye Research*.

[39] Ma X., Zhang H., Pan Q. (2013). Hypoxia/aglycemia-induced endothelial barrier dysfunction and tight junction protein downregulation can be ameliorated by citicoline. *PLoS ONE*.

[40] Giannotta M., Trani M., Dejana E. (2013). VE-cadherin and endothelial adherens junctions: active guardians of vascular integrity. *Developmental Cell*.

[41] Vestweber D., Winderlich M., Cagna G., Nottebaum A. F. (2009). Cell adhesion dynamics at endothelial junctions: VE-cadherin as a major player. *Trends in Cell Biology*.

