# Supplementary material for: Keratinocyte Growth Factor-2 Is Protective in Oleic Acid-Induced Acute Lung Injury in Rats
Source: Evid Based Complement Alternat Med. 2019 Jul 15;2019:9406580. doi: 10.1155/2019/9406580 (PMC6662415; doi:10.1155/2019/9406580)
Supplement: Supplementary Materials — This support material focuses on disease ALI/ARDS and target location for intervention in this study as well as supplemental experiments (ELISA for TNF-a/IL-10). [file 9406580.f1.pdf]

This support material focuses on disease ALI/ARDS and target location for intervention in this study as well as supplemental experiments (ELISA for TNF-a/IL-10).

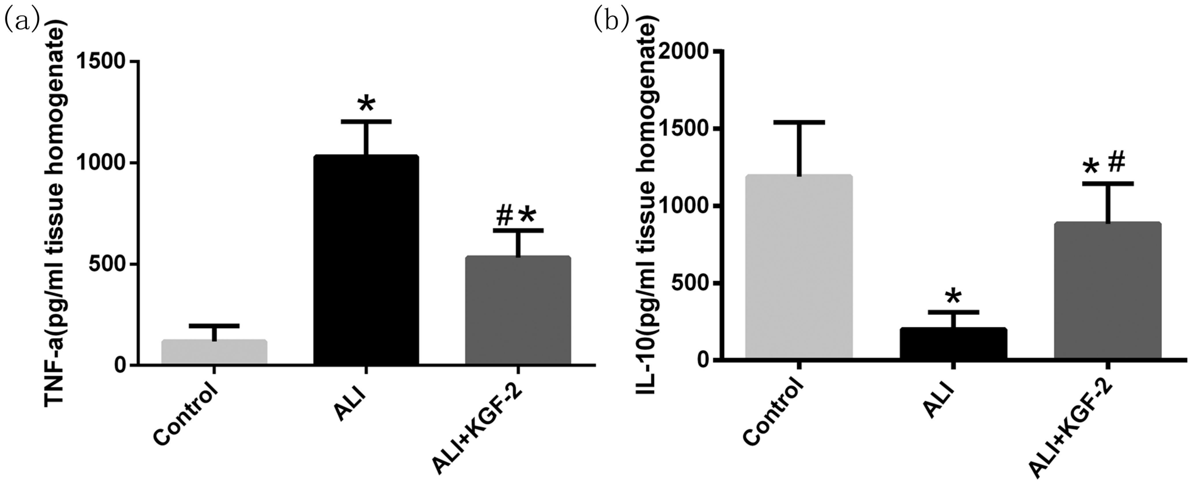

FIGURE : shows a significant increase in TNF-a value in the ALI group compared with the control group, and improved after KGF-2 intervention; shows a significant reduce in IL-10 value in the ALI group compared with the control group, and improved after KGF-2 intervention (a)Changes of TNF-a expression in lung tissue. (b)Changes of IL-10 expression in lung tissue. (*< 0.01 versus control group; #<0.01 versus ALI group).
